# Creative thinking and cognitive estimation in Parkinson’s disease

**DOI:** 10.1186/s42466-023-00304-1

**Published:** 2024-02-15

**Authors:** Marcus Heldmann, Celia Rinckens, Norbert Brüggemann, Mohamed Al-Khaled, Thomas F. Münte

**Affiliations:** 1 Department of Neurology, University of Lübeck, Lübeck, Germany; 2 Center for Brain, Behavior and Metabolism, University of Lübeck, Ratzeburger Allee 160, 23538 Lübeck, Germany

**Keywords:** Parkinson’s disease, Divergent thinking, Convergent thinking, Creativity, Cognitive estimation, Dopamine

## Abstract

**Background:**

Patients with Parkinson’s disease (PD) have been reported to exhibit unusual bouts of creativity (e.g., painting, writing), in particular in the context of treatment with dopaminergic agents. Here we investigated divergent and convergent thinking thought to underlie creativity. In addition we assessed cognitive estimation.

**Method:**

Twenty PD patients and 20 matched healthy control participants were subjected to the Guilford Alternate Uses task (divergent thinking), the remote associates task (convergent thinking) and two tests of cognitive estimation.

**Results:**

No group differences were found for the convergent thinking task, while the Guilford Alternate Uses task revealed a decreased number of correct responses and a reduced originality for PD patients. Originality in PD was correlated to total daily dose of dopaminergic medication. Moreover, both tasks of cognitive estimation showed an impairment in PD.

**Conclusion:**

Only minor effects were found for psychometric indices of subprocesses of creative thinking, while estimation, relying on executive functioning, is impaired in PD. We suggest to take a product oriented view of creativity in further research on altered creative processes in PD.

## Introduction

The neurochemical hallmark of Parkinson’s disease is the loss of dopaminergic neurons in the nigrostriatal pathway, which is counteracted by the application of dopaminergic medication, most often L-Dopa or direct dopamine agonists.

One of the many facets of dopaminergic action is its apparent influence on creativity. For example, taking spontaneous eye-blink rate (EBR) as a proxy for dopaminergic tone, Chermahini and Hommel [13] found that EBR differentially predicted flexibility in divergent and convergent thinking, both viewed as elements of creativity. More direct evidence comes from a number of behavioural genetics studies investigating the relationship of dopaminergic polymorphisms to divergent thinking [39, 40, 45, 51, 52, 68] and cognitive flexibility [16, 22, 23], another key element of creativity. Importantly for the present investigation, a number of case reports have linked bouts of creative activity such as painting or poetry writing with the initiation of dopaminergic activity [12, 29, 34, 36, 56, 57, 63, 67].

### Operationalization of creativity

Creativity is a complex trait and any attempt to define or measure creativity is bound to be incomplete. Stein, in the preface of his 1974 book on “Stimulating creativity”, proposed a product-oriented definition of creativity: “Creativity is a process that results in a novel work that is accepted as useful, tenable, or satisfying by a significant group of people at some point in time” [61]. By contrast Newell et al. [46] took a process-oriented view and stated that, even though its products sometimes are extraordinary, creative thinking basically is the same as the thinking involved in solving ordinary problems. While the literature on creativity is still undecided with regard to these views, we initially take a product-oriented view in this work.

The quantification of creativity is not without difficulties, as the very nature of creative minds is to think out of the box and to come up with new ideas, whereas the nature of psychometric quantification is to test people in standardized settings. These difficulties notwithstanding, divergent and convergent thinking have been identified as key aspects of creativity which can be quantified [44]. Divergent thinking has been identified as the ability of a person to generate original ideas within a given time-period. This is quantified, for example, by the Guilford’s Alternative Uses Task [27] which requires participants to list as many possible uses for common household objects (such as a brick, a paperclip, a newspaper). The test provides different measures (Originality, i.e. statistical novelty; Fluency, i.e. total number of responses; Flexibility, i.e. number of categories responses are drawn from; Elaboration; amount of detail given) to characterize creativity. Laboratory measures of divergent thinking are correlated to measures of everyday creative activities as measured by questionnaires [68, 69] as well as creative achievements [30] but differences have also been reported [26].

Divergent thinking assesses the ability to come up with multiple, unrestricted solutions for an ill-defined problem, whereas convergent thinking, in turn, requires the generation of one particular solution of a particular problem. This is often tested by the remote associates task (RAT) first described by Mednick [41, 42] or by some version of this task. In the RAT, participants are confronted with three words that are connected by one solution word. Importantly, the association of the three words with the solution can vary. For example, the triplet “same/tennis/head” is associated with the solution “match” by either being synonymous (same = match), by forming a compound noun (matchhead), or by having a close semantic association (tennis match). Problem solving in this task requires creativity, as participants need to dismiss and suppress incorrect solutions and to think about remotely related words. Results on the RAT correlate highly with success on other types of insight problems [55].

### The present study

To assess the effect of PD and dopaminergic medication on creativity, we applied tasks assessing divergent (Guilford Alternate Uses Task) and convergent thinking (a German version of the RAT) to a group of 20 patients with PD and a group of matched control participants. We expected a relationship of indices of creativity to the total daily dosage of dopaminergic medication in PD. With regard to group differences, we had no firm expectations.

Moreover, we employed several tests aimed at cognitive estimation. Such tests are viewed as measures of reasoning and self-monitoring [61]. They require participants to answer questions for which the answer is not immediately available and must therefore be estimated, e.g. “How many camels live in the Netherlands?” It is further thought that cognitive estimation relies on regions of the frontal lobes which in turn are part of the fronto-striatal-thalamic loops which are compromised in PD. We have selected cognitive estimation, as it requires deliberative strategies that have certain similarities with the ones also enlisted in divergent thinking. Previous results on cognitive estimations with regard to PD have been mixed, however. While Bullard et al. [9] found significantly poorer estimation performance in demented PD patients compared to controls, Appollonio et al. [2] did not find consistent estimation deficits in their group of non-demented PD. D’Aniello et al. [17] pointed out that only a relatively small number of non-demented medicated PD patients perform below the cut-off on cognitive estimation and later the same group reported that PD patients had mainly problems with length-related estimations.

In light of mixed results of previous studies on creativity, we had no firm hypothesis with regard to differences between patients and healthy controls.

## Methods

Twenty PD patients were recruited from the outpatient movement disorders clinic of the Dept. of Neurology, University Hospital Schleswig–Holstein. Inclusion criteria were idiopathic PD as diagnosed by an experienced neurologist according to the UK Brain Bank criteria and lack of clinical depression (score < 12 on the Beck Depression Inventory II, BDI II) [5] as well as lack of dementia (score > 18 on the Parkinson Neuropsychometric Dementia Assessment, PANDA) [32]. Patients were tested in the on-state. Motor status and severity of the disease were assessed by the Unified Parkinson’s disease rating scale III (UPDRS III) [25] and Hoehn and Yahr scale (H&Y scale) [28]. The L-Dopa equivalent daily dosage (LEDD) was calculated using the method of Schade et al. [54]. Only medicated PD patients were included. Patients with deep brain stimulation were excluded.

Twenty-one control participants were recruited and matched for age, educational status and gender. These reported no past or present neurological or psychiatric illnesses. The demographic characteristics of the participants are given in Table 1.

**Table 1:** Characteristics of participants

	PD (n = 20)	HC (n = 21)	PD versus HC (*p* value)	Effect size, Cohen’s d
Women/Men	10/10	11/10	n.s	
Age (years)	68.1 (9.9)	67.3 (7.3)	n.s	
Education (years)	12.2 (2.9)	13.0 (3.0)	n.s	
Total LEDD (mg)	570 (311)	–	–	
UPDRS part III	18.1 (10.9)	–	–	
Disease duration (years)	7.15 (5.5)	–	–	
PANDA	23.93 (4.1)	25.1 (3.6)	n.s	0.30
BDI-II	9.4 (4.9)	5.8 (3.9)	0.014	0.81
RWT „M”^a^	53.7 (31.5)	55.9 (29.9)	n.s	0.07
RWT „G-R”^a^	48.3 (29.3)	58.9 (27.7)	n.s	0.37
RWT „Food items “^a^	45.8 (26.3)	66.8 (23.7)	0.01	0.84
RWT „Sports-Fruits “^a^	55.2 (23.4)	69.1 (24.3)	n.s	0.58
FWIT (interference)^b^	54.7 (8.4)	61.2 (10.6)	0.038	0.68
CVLT List A (total)^a^	15.5 (26.5)	28.3 (24.6)	n.s	0.50
CVLT List B (total)^a^	21.9 (21.5)	31.1 (27.7)	n.s	0.37
CVLT delayed free recall^a^	22.8 (28.6)	22.3 (21.6)	n.s	0.02
CVLT cued recall^a^	20.2 (23.8)	31.4 (22.9)	n.s	0.47
LPS 3^a^	73.6 (19.6)	64.9 (27.6)	n.s	0.36
LPS 4^a^	79.6 (17.8)	73.4 (23.5)	n.s	0.29
TAP alertness without warning^c^	337 (82)	285 (61)	0.03	0.72
TAP alertness with warning^c^	305 (74)	274 (55)	n.s	0.51
TAP Go/noGo^c^	464 (70)	428 (72)	n.s	0.50
TAP Go/noGo, #errors^d^	1.4 (1.3)	2.1 (2.4)	n.s	0.014

^a^Based on percentiles according to published norms

^b^Based on T-values according to published norms

^c^Milliseconds

^d^Kruskal–Wallis-test (effect size eta^2^)

*n.s.* Non-significant

### Neuropsychological assessment

To characterize the cognitive status of the participants, a number of neuropsychological tests were performed. Global cognitive function was assessed by the PANDA [32], a test battery developed for the assessment of cognition in PD. Verbal fluency as one facet of executive functions was assessed with the Regensburger Wortflüssigkeits Test (RWT; lexical fluency for the letter “M”, lexical flexibility by “G” and R” alternating, semantic fluency for category “food”, semantic flexibility by testing “sports” and “fruits” alternating) [3]. Further, the German version of the Stroop test (Farb-Wort Interferenztest, FWIT) [4] was applied to assess susceptibility to interference. Verbal learning and memory was evaluated by the German version of the California Verbal Learning Test (CVLT; immediate and delayed free and cued recall) [47]. Reasoning was assessed by subtests 3 and 4 from the “Leistungs-Prüfsystem” (LPS3 and LPS4) [62]. Attention functions were tested using a computerized test-battery (Testbatterie zur Aufmerksamkeitsprüfung, TAP) [70], of which the subtests Alertness and Go/Nogo were selected.

### Tests of creativity

#### Remote associates task

The RAT aims at measuring creative thought without requiring prior knowledge. In the US two versions of the test of 30 items each were developed [41, 42] but more recently Bowden and Beeman [7] have published normative data for 144 English problems. At the time of the planning of this study, no German materials were available. Such materials have been published in the meantime [35], however. We thus created our own set of problems following the procedures described in Bowden and Beeman [7].

A list of 30 RAT problems created by one of the authors (CR) either anew or by translating English materials was tested in 50 student volunteers. A final list of 10 problems was selected. The participants were informed that the solution word could come before or after the words mentioned and that it could not be exclusively nouns but words of all categories. The subjects were asked to find the correct association within a maximum time of 30 s. The two groups were compared on the basis of the number of correct solutions and the solution time.

#### Alternate uses task

The original task (as available from www.mindgarden.com) was used. Participants were asked to list as many possible uses for three common household items (brick, shoe, and newspaper) as they can within 10 min. Scoring was done according to four aspects as suggested by the scoring instructions:

Originality: The uniqueness of responses was scored following Wallach and Kogan [64]. Basically, it is checked whether an answer has also been given by another participant. If an item is original in this sense, one point is awarded.

*Fluency* The total of all non-redundant responses.

*Flexibility* The number of different categories used. For this measure responses were sorted into different conceptual categories. For the stimulus “newspaper”, for example, the response “to hit somebody on the head” would be assigned to the category “weapon”, whereas the response “to start a fire with” would be assigned to the category “fuel”.

*Elaborateness* This measure pertains to the level of detail of the given items which usually are written down by the participants. As PD patients tend to be slow to write and also exhibit micrography, we decided to record the answers by the experimenter. As inadvertent paraphrasing might have occurred, we refrained from using this metric.

*Normalized web distance* We also obtained the mean normalized web distance (NWD) of all responses given by a participant. We adopted the method of Cilibrasi and Vitányi [14], which is illustrated by an example given by these authors. At the time of their study, a Google search for the word “horse” yielded 46,700,000 hits, while the number of hits for the term “rider” was 12,200,000. Next, the combined search of “horse” and “rider” returned 2,630,000 pages. Finally, Google indexed a total of 8,058,044,651 web pages at the time of the experiment. Applying the formula given by Cilibrasi and Vitányi [14] the normalized web distance between the terms “horse” and “rider” is eG(horse, rider) ≈ 0.443

$$\begin{aligned} eG\left( {x,y} \right) & = \frac{{G\left( {xy} \right) - \min \left\{ {G\left( x \right),G\left( y \right)} \right\}}}{{\max \left\{ {G\left( x \right),G\left( y \right)} \right\}}} \\ & = \frac{{\max \left\{ {\log f\left( x \right),\;\log f\left( y \right)} \right\} - \log f\left( {x,y} \right)}}{{\log N - \min \left\{ {\log f\left( x \right),\;\log f\left( y \right)} \right\}}} \\ \end{aligned}$$

where f(x) is the number of pages containing x, f(y) is the number of pages containing y and f(x, y) is the number of pages containing both x and y.

#### Cognitive estimation tasks

We used two tasks to assess cognitive estimation abilities. First, we used a German version of the Cognitive Estimation Task (CET). The (CET) was originally devised by Shallice and Evans [59] as a test of an individual’s ability to provide appropriate cognitive estimates. They first reported that patients with damage to the frontal lobes not only performed poorly on the CET but in addition were also likely to produce bizarre over- or under-estimates. Besides frontal lobe damage, CET scores were also significantly impaired in a variety of disorders such as Alzheimer’s disease [18, 50], frontotemporal dementia [43] and vascular dementia [6]. Please note, that previous studies on Parkinson’s disease patients have been inconsistent with some studies reporting abnormal findings (e.g., [53]) while another finding no impairment [2].

Besides intact reasoning abilities, solutions of CET items are also dependent upon intact and retrievable semantic knowledge. For example, to provide an appropriate answer for the item “How many camels are there in Holland?” it is necessary to access the semantic knowledge about camels kept in zoos, circuses and elsewhere as well as information about Holland.

Second, we also employed the *Test zum kognitiven Schätze*n (TKS) [8]. The TKS comprises 16 questions, four for each of the four dimensions size, weight, quantity (requiring to estimate the specific attribute instantaneously from stimulus pictures), and time (e.g., ‘How long does a flight from Frankfurt to New York take?’). This test requires estimation from visual input or prior knowledge, rather than deductive reasoning which is required by the CET. The authors claim that the improvement of the TKS compared to the CET consists in the introduction of the four separately recorded estimation dimensions size, weight, number and time as well as in the abandonment of non-numerical dimensions, such as knowledge questions, as these have been criticized with regard to the CET. On the other hand, the TKS does not tax reasoning abilities to the same extent as the CET. We therefore included both tests.

### Statistical analysis

In the present study, we adopt the concept of "Descriptive Data Analysis" (DDA) as defined by Abt [1]. Confirmatory Data Analysis (CDA) encounters challenges in randomized comparative ("controlled") studies featuring numerous variables. These challenges stem from the proliferation of desired inferential statements, leading to excessively stringent adjusted significance levels ("Bonferronization"). To bridge the conceptual gap between CDA and exploratory data analysis, Abt [1] suggests DDA. Consequently, we performed t-tests for the different measures and also Spearman correlations as appropriate.

## Results

### Background neuropsychological tests

Results from the neuropsychological background test-battery are shown in Table 1. As we aimed to include only non-demented PD patients, the PANDA cog score did not differ between PD and HC groups. There was a slight but significant increase of depressive symptoms in the PD group (BDI-II). While the PD group generated fewer words in the verbal fluency task (RWT), this was significant only for the categorical fluency condition “food”. The two additional executive function tasks (LPS3 and LPS4) did not reveal differences between groups. The memory test (CVLT) was not different between groups. Finally, while reaction times were slower in the PD group for the attention tests, this was significant only for the condition without warning tone of the alertness test.

### Alternate uses task

**Fig. 1:**
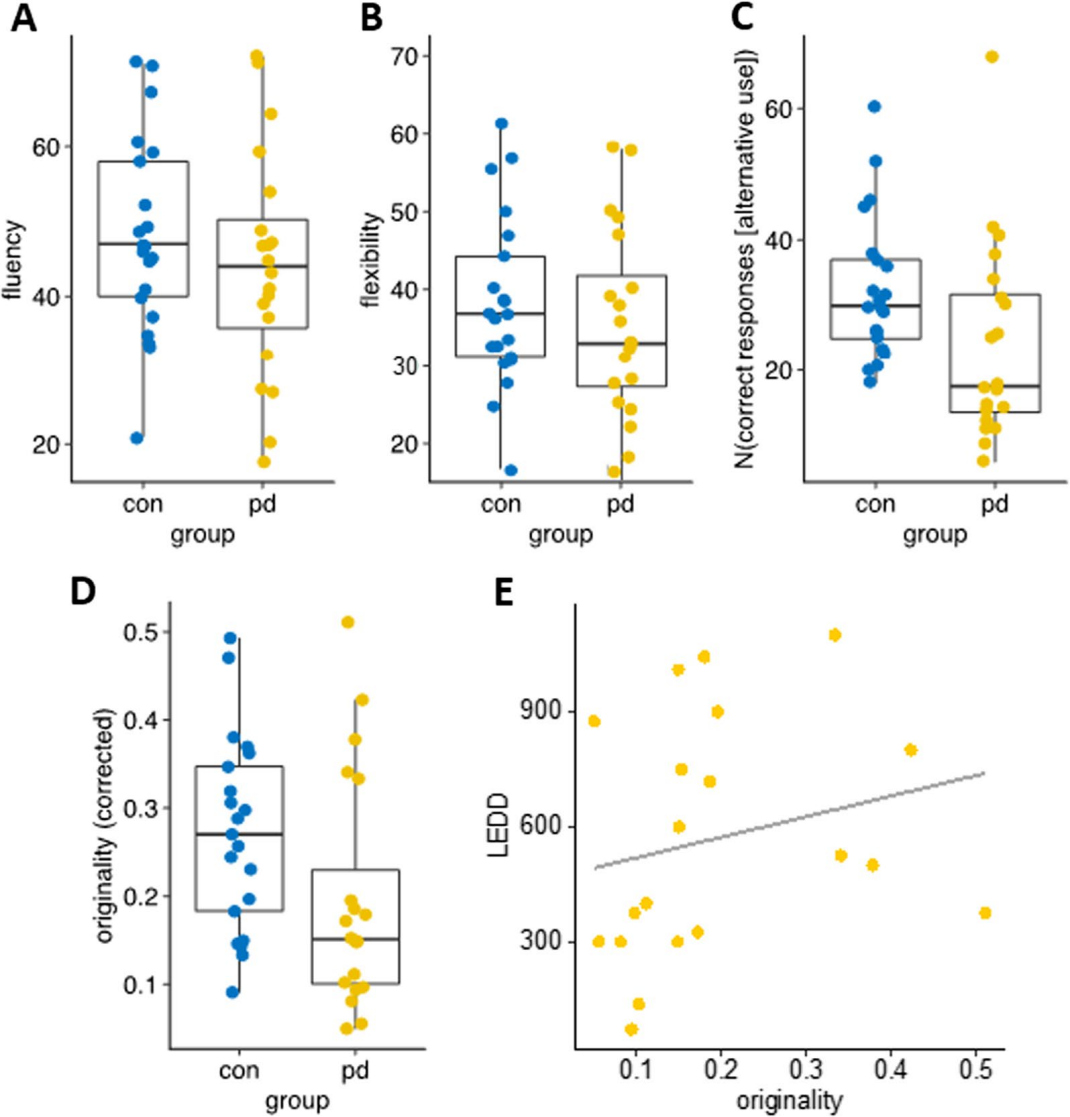
Results of the Alternate Uses Task: fluency (**A**) and flexibility (**B**) measures were not different between groups. There were less correct responses **C** in PD and originality of responses **D** was reduced in patients. In PD, there was a tendency to show more original responses with higher dopaminergic medication (**E**)

*Fluency* The evaluation is based on the sum of all answers per respondent. It should be noted that the values deviate from the values in the literature due to the completion of two examination forms to increase the reliability of the measurement. There was only a slight, non-significant difference between the two groups [t(39) = 0.91, *p *= 0.368; Fig. 1]. The Spearman correlation between the fluency measure and either LEDD (R = 0.21, n.s.) or UPDRS III (R =  − 0.08, n.s.) was not significant.

*Flexibility* The number of different categories of possible uses was scored. There was a tendency for PD patients to use less categories [Fig. 1b; t(39) = 1.73, *p *= 0.091]. There was no correlation to either LEDD or UPDRS III (both *p *> 0.24).

*Number of correct answers* In addition to the total number of responses, the correct answers were scored (agreement of 2 investigators). Comparison of the two groups showed a significant difference between the two groups in the number of items scored as correct. The effect size can be classified as moderate [t(39) = 2.05, *p *= 0.047, Cohen’s d = 0.64].

*Originality* PD patients were significantly less original than HC participants [PD mean/SD = 0.195/0.120; HC = 0.271/0.102; t(39) = 2.16; *p *= 0.036; Cohen’s d 0.65]. Importantly, in PD patients originality was correlated to LEDD (R = 0.45; *p *= 0.046) with higher LEDD related to greater originality. This correlation was also present, when only the dosage of dopamine agonists were used (based on 18 patients as two patients did not receive dopamine agonists; R = 0.49; *p *= 0.039). No correlation was found between originality and UPDRS-III.

*Normalized Web Distance* The semantic proximity of the answers was assessed by calculating the normalized web distance using the Google platform as detailed above [14]. There was no difference between groups with respect to all responses, correct responses and original responses [all t(39) < 1.01; all *p *> 0.31].

### Remote associate’s task

The ability of participants to come up with a word connecting the three words of a triplet within 15 s is compared. The percentage of solved puzzles was very similar for PD patients (mean/SD = 63.5/16.71) and the HC group [63.57/15.18; t(39) = 0.014, *p *= 0.988].

### Cognitive estimation test

The deviation score was assessed and differed significantly between PD patients (mean/SD = 5.78/3.02) and the HC group [mean/SD = 3.05/3.59; t(39) = 2.64, *p *= 0.011]. Larger deviation scores indicate worse performance.

### Test zum Kognitiven Schätzen (TKS)

**Fig. 2:**
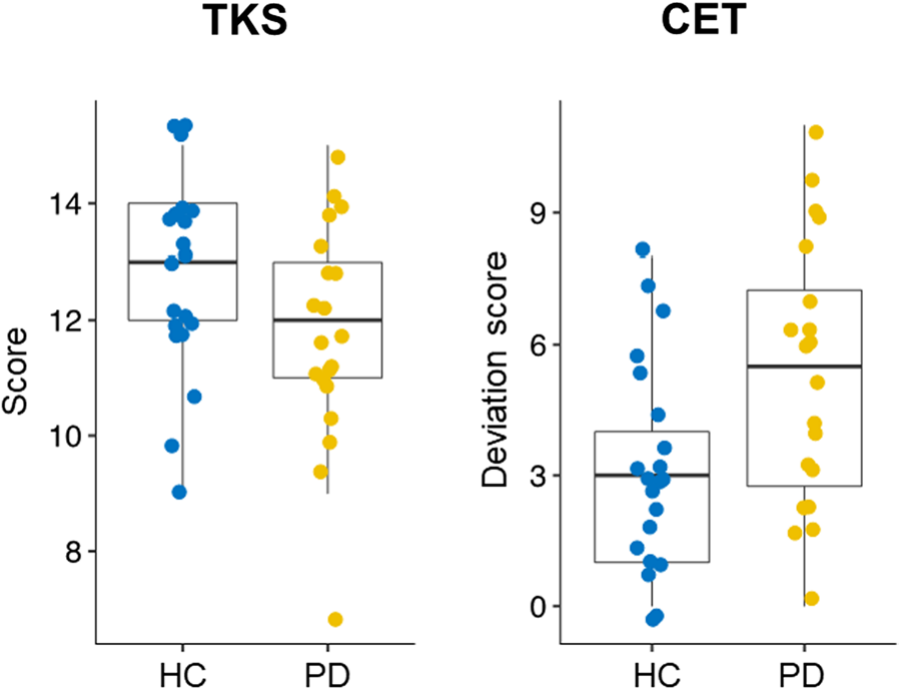
Both estimation tests showed worse performance in PD. The total score in the TKS was reduced (left) and the deviation score in the CET was increased (right)

The total test score differed between PD patients (mean/SD = 11.75/1.88) and HC [12.86/1.53; t(39) = 2.08, *p *= 0.044]. Higher scores indicate better estimation abilities (Fig. 2).

## Discussion

In this group of non-demented PD patients we found evidence for an impairment of divergent thinking (as indicated by the Alternate Uses Task) in that PD patients produced less correct answers and less original answers than healthy matched control participants. Interestingly, the degree of originality was correlated to the daily dosage of dopaminergic medication. There were no differences with regard to convergent thinking as measured by the Remote Associates Task. Finally, there was evidence for impaired abilities in two tests of cognitive estimation.

### PD and process-oriented creativity

In this work we followed a process-oriented definition of creativity [46], for which divergent and convergent thought processes are deemed to be important [44]. The reduced number of correct answers and reduced originality in the Alternate Uses Task speaks for a reduced ability for divergent thinking, i.e. the ability to come up with novel information, in PD. This might be expected given that creativity has been associated with the dopaminergic system and PD is characterized by a hypodopaminergic state. The fact that in our PD group originality was correlated to the daily dosage of dopaminergic drugs supports this interpretation as originality is partially restored by these medications.

A similar study was performed by Faust-Socher et al. [21]. In their study PD patients performed better than controls in the TACT battery, comprising of visual and verbal subtests divergent thinking tasks similar to the Alternate Uses Task. Specifically, patients showed a slightly increased number of responses and also more original responses. Interestingly, the quality of responses was increased in patients with high LEDD (mean 961 mg/d) compared to those with low LEDD (148 mg /d). These results dovetail nicely with the current study and suggest a relationship between dopaminergic medication and originality in divergent thinking task. Studies in de novo patients should be conducted and we suggest that the hypodopaminergic state in these patients should lead to reduced divergent thinking.

It is noteworthy that both, the current and the Faust-Socher et al. [21] study, did not find any alteration of convergent thinking in PD as assessed by the RAT.

One problem with a process-oriented operationalization of creativity is that this approach might measure processes that are related to creativity but rather in the sense of being a necessary precondition. In the current investigation, we neither measured variables related to the person (personality traits, genetic traits, c.f. [20, 33, 66] nor did we measure product related facets of creativity. The problem is that creativity as described in case reports comes from the product. Canesi et al. [11] have tried to address the question of whether or not dopaminergic treatment might act differentially in PD patients with previous artistic inclinations and PD patients without such prior activities. Creativity was lowest in PD patients without prior artistic activities in spite of dopaminergic treatment. The authors concluded that their results do not support a relationship between DT and the emergence of artistic creativity. They also call for investigations of creative thinking on and off medication. Taken together, the Canesi et al. [11] results suggest that personal trait are key for artistic productivity.

### PD and product-oriented creativity

Defining creativity in a product-oriented fashion, Lhommée et al. [37] identified creative PD patients (n = 11) as well as non-creative PD patients selected for deep brain stimulation (DBS) to alleviate motor systems. Creative patients produced works of art, i.e. sculptures (1 patient), face casts (1 patient), paintings (3 patients), glass paintings (1 patients), drawings (1 patient) and writings (3 patient). The fact that artistic production had either started or was exacerbated upon initiation of dopaminergic therapy suggested a relationship of dopaminergic therapy to the production of works of art. Patients were assessed prior and 1 year after surgery. DBS allowed for a reduction of dopaminergic medication by 68% (similar in creative and non-creative groups). Interestingly, only 1 of the 11 creative PD patients remained creative after surgery. This is a clear indication that creative activity is driven by dopaminergic medication.

This is corroborated by a case series reported by Garcia-Ruiz et al. [24] comprising 21 patients (20 PD, 1 restless-legs syndrome) with enhanced creativity (painting, building scale models, etc.). All patients had started to engage in artistic activities after initiation of dopaminergic treatment, mostly dopamine agonists (DA, pramipexole, 14/21; ropinirole, 4/21; rotigotine 2/21). Of all DAs, pramipexole and ropinirole have been reported to be most frequently associated with impulse control disorders (ICD) which may be due to their preferential affinity for the D3 receptor [58]. The question therefore arises, as to whether or not enhanced creativity might be viewed as yet another instance of impulsive compulsive behaviours that occur quite frequently in PD patients treated with dopamine agonists [65]. Some authors have therefore viewed newly developed creative activities such as writing or painting as having the same etiological background as typical impulse control disorders and punding behavior (e.g., [38]). Canesi et al. [10] specifically addressed the association between creativity and ICDs in PD patients with or without increased artistic-like production (defined by producing any form of art for more than 2 h/day) and healthy controls. Measures of creativity were not correlated with scores on the Minnesota Impulsive Disorders Interview thus suggesting that artistic-like production in the course of dopaminergic treatment might have a different background than ICDs. On the other hand, a questionnaire study on about 300 PD patients did report an association with newly developed creativity and ICDs [31].

### Cognitive estimation

Cognitive estimation tasks assess an individual’s ability to produce rough estimates in response to questions for which the exact answer is not known [59]. Cognitive estimation is thought to engage executive functions and bizarre answers are obtained in patients with frontal lobe damage [15, 59].

Previous research in cognitive estimation has not provided an unanimous consensus regarding whether PD leads to diminished cognitive estimation abilities when compared to healthy individuals. A number of studies involving PD patients have concentrated on the estimation of time intervals with both non-demented medicated [48, 60] and non-medicated [49] PD patients showing significantly poorer performance compared to their healthy counterparts.

In the present investigation we employed two tests of estimation. Concerning the CET, Bullard et al. [9] observed that individuals with demented PD provided significantly less accurate estimates than controls for items related to weight and quantity but not for those involving time or distance. However, for non-demented PD individuals, Appollonio and colleagues [2] did not identify any notable deficits in CET performance. More recent research has reported cognitive estimation deficits in non-demented medicated PD patients [17, 53], albeit pathological estimation abilities were observed only a small percentage of individuals. In the present investigation, we found increased deviation scores in the German version of the CET in PD patients compared to HC. However, inspection of the distribution of the data shows considerable overlap of both groups. The Test zum Kognitiven Schätzen (TKS) involves estimation tasks involving height, weight, number and time dimensions, which are thought to underlie many everyday activities. The TKS does not involve deductive reasoning to the same extent as the CET. Again, we found slight but significant impairment of the PD patients on the group level with considerable overlap of the distributions of the two samples.

### Limitations

The current study is not without limitations. First, as with other similar studies, the sample size is rather small. Thus, small effects might have gone undetected. Moreover, to pinpoint the possible role of dopaminergic medication, in particular dopamine agonists, a comparison of patients in the on and off state might have been informative (c.f. [11]). However, the resulting motor impairment in the off state might overshadow any genuine effect on creative thinking. An alternative would be the investigation of creative thinking in unmedicated de novo patients. Finally, future studies should also include an assessment of artistic activity for two time points (prior to disease onset, current situation) in order to focus also on product aspects of activity. A possible instrument is the Inventory of Creative Activities and Achievements (ICAA [19]).

## Conclusion

In conclusion, our study showed slight impairments in a test of divergent thinking in PD regarding the number of correct responses as well as the originality of responses. A standard test of convergent thinking did not reveal deficits in PD. These tests, while they have been linked to creative processes, do not appear to assess creativity in the sense of production of art. Thus, we advocate to adopt a product-oriented view of creativity in further research of creative bouts in PD. Moreover, the study also revealed slight impairments of PD with regard to cognitive estimation corroborating earlier results.

## Data Availability

Data will be made available upon reasonable request.
